# Clinical predictors of long-term survival in newly diagnosed transplant eligible multiple myeloma — an IMWG Research Project

**DOI:** 10.1038/s41408-018-0155-7

**Published:** 2018-11-23

**Authors:** Saad Z. Usmani, Antje Hoering, Michele Cavo, Jesus San Miguel, Hartmut Goldschimdt, Roman Hajek, Ingemar Turesson, Juan Jose Lahuerta, Michel Attal, Bart Barlogie, Jae Hoon Lee, Shaji Kumar, Stig Lenhoff, Gareth Morgan, S. Vincent Rajkumar, Brian G. M. Durie, Philippe Moreau

**Affiliations:** 1 Hematogic Oncology and Blood Disorders, Levine Cancer Institute/Atrium Health, Charlotte, NC United States; 2 Cancer Research and Biostatistics, Seattle, Washington United States; 3 Seragnoli Institute of Hematology, Bologna University School of Medicine, Bologna, Italy; 4 Centro de Investigación Médica Aplicada, IDISNA, CIBERONC, Clinica Universidad de Navarra, Pamplona, Spain; 5 Department of Internal Medicine V, University Hospital Heidelberg, Heidelberg, Germany; 6 University Hospital Ostrava and the Faculty of Medicine, CRAB, University of Ostrava Adam Rosenthal, Seattle, United States; 7 Department of Hematology, Malmo University Hospital, Malmo, Sweden; 8 Hospital Universitario 12 de Octubre, Madrid, Spain; 9 Service d’Hématologie, CHU de Toulouse-Institut Universitaire du Cancer de Toulouse Oncopole, Toulouse, France; 10 Hematology, Mount Sinai University, New York, United States; 11 Gachon University Gil Medical Center, Gachon University School of Medicine, Incheon, Republic of Korea; 12 Department of Internal Medicine, Division of Hematology, Mayo clinic, Rochester, MN United States; 13 Skane University Hospital, Lund, Sweden; 14 MIRT, UAMS, Myeloma Insitute, Little Rock, United States; 15 Division of Hematology, Mayo Clinic, Rochester, MN United States; 16 Hematology/Oncology, Samuel Oschin Comprehensive Cancer Institute, Cedars-Sinai Medical Center, Los Angeles, United States; 17 service d’Hematologie, CHU de Nantes, Nantes, France

## Abstract

Purpose: multiple myeloma is considered an incurable hematologic cancer but a subset of patients can achieve long-term remissions and survival. The present study examines the clinical features of long-term survival as it correlates to depth of disease response. Patients & Methods: this was a multi-institutional, international, retrospective analysis of high-dose melphalan-autologous stem cell transplant (HDM-ASCT) eligible MM patients included in clinical trials. Clinical variable and survival data were collected from 7291 MM patients from Czech Republic, France, Germany, Italy, Korea, Spain, the Nordic Myeloma Study Group and the United States. Kaplan–Meier curves were used to assess progression-free survival (PFS) and overall survival (OS). Relative survival (RS) and statistical cure fractions (CF) were computed for all patients with available data. Results: achieving CR at 1 year was associated with superior PFS (median PFS 3.3 years vs. 2.6 years, *p* < 0.0001) as well as OS (median OS 8.5 years vs. 6.3 years, *p* < 0.0001). Clinical variables at diagnosis associated with 5-year survival and 10-year survival were compared with those associated with 2-year death. In multivariate analysis, age over 65 years (OR 1.87, *p* = 0.002), IgA Isoty*p*e (OR 1.53, *p* = 0.004), low albumin < 3.5 g/dL (OR = 1.36, *p* = 0.023), elevated beta 2 microglobulin ≥ 3.5 mg/dL (OR 1.86, *p* < 0.001), serum creatinine levels ≥ 2 mg/dL (OR 1.77, *p* = 0.005), hemoglobin levels < 10 g/dL (OR 1.55, *p* = 0.003), and platelet count < 150k/μL (OR 2.26, *p* < 0.001) appeared to be negatively associated with 10-year survival. The relative survival for the cohort was ~0.9, and the statistical cure fraction was 14.3%. Conclusions: these data identify CR as an important predictor of long-term survival for HDM-ASCT eligible MM patients. They also identify clinical variables reflective of higher disease burden as poor prognostic markers for long-term survival.

## Introduction

The last decade has witnessed major progress in clinical outcomes in multiple myeloma (MM), attributable to the introduction of several novel agents which, when combined with either each other or conventional cytotoxic drugs, have imparted a high frequency of complete responses (CR)^1–4^. This is especially true for younger and high-dose melphalan-autologous stem cell transplant (HDM-ASCT) eligible patients where pre-transplant induction therapy is able to provide > 60% rates of very good partial response or better. HDM-ASCT further deepens the response and results in ~CR rates. Several prospective studies have reported strong correlation between depth of response (achieving CR) and progression-free survival (PFS) and overall survival (OS) in the era of novel agents. There is also a clear recognition that patients achieving CR may have variable PFS/OS^5^, perhaps dictated by differences in disease biology and shortcomings of the current response criteria. Minimal residual disease (MRD) testing, by either flow or DNA sequencing, has demonstrated that CR patients may have residual disease (MRD positive) putting them at risk for early relapse^6^.

These advances have re-ignited the debate on possible functional curability of a subset MM patients^5^. The Spanish Myeloma group recently reported the strong correlation of depth of response to higher likelihood of long-term PFS/OS (12-year PFS was 28% in CR, 19% in nCR, 10% in VGPR and 11% in PR groups; 12-year OS was 35% in CR, 22% in nCR, 16% in VGPR and 16% in PR groups) among 344 post HDM-ASCT patients followed for a median of nearly 13 years^7^. The current study is a collaborative effort by the Internal Myeloma Working Group to closely examine the clinical predictors of long-term survival in MM (>10 yr) in terms of their presenting features, quality of response and management in order to guide therapeutic investigations aimed at long-term disease control.

## Patients and methods

### Patient population

A total of 7291 patients with survival data were considered for the analysis, age limit up to 75 years. These patients come from the following nations: Czech Republic, France, Germany, Italy, South Korea, Spain, the Nordic Myeloma Study Group (Sweden, Denmark, Norway), and the United States. Tables comparing patient characteristics for several key variables for patients meeting specified characteristics are included, as are tables showing a summary of available data by country (Supplemental Table 3). It is important to note that over 90% of the patients in the dataset were from the pre-novel therapy induction era and ~10% did received thalidomide as part of their upfront therapy (Total Therapy 2 thalidomide arm, GMMG-HD3 thalidomide arm and BO2002).

### Statistical analysis

Kaplan–Meier curves^8^ were used to assess OS, PFS, and CR duration (where applicable). Relative survival (RS)^9^ was computed for all patients with available data. The goal of RS analysis is to compare the survival of a cohort with a certain disease to the survival of the general population with similar characteristics to that cohort. We estimate the ratio of observed survival amongst patients with a certain condition to expected survival in a comparable population with comparable characteristics (such as nationality, age, and sex) to those patients. In this analysis, we consider a patient’s nationality, age at enrollment, and sex (where available), and obtained expected survival estimates from life tables for a person with those characteristics starting in the year of the patient’s enrollment (note: where sex data not available, we use data from life tables estimated for nationality and age which did not adjust for sex). Here, we provide estimates for RS for the IMWG data overall and by individual country, with point estimates at each 1-year interval from start of therapy, and a 95% confidence band for the RS.

Berkson–Gage cure modeling^10^ was performed for a proportion of patients who represent a “cure proportion;” in other words, these patients are said to have survival according to the normal survival rates for a person of that age/sex/nationality. The figures in the manuscript represent survival curves, where survival is the survival for the “cure proportion” plus the survival for the remainder of patients.

## Results

**Fig. 1:**
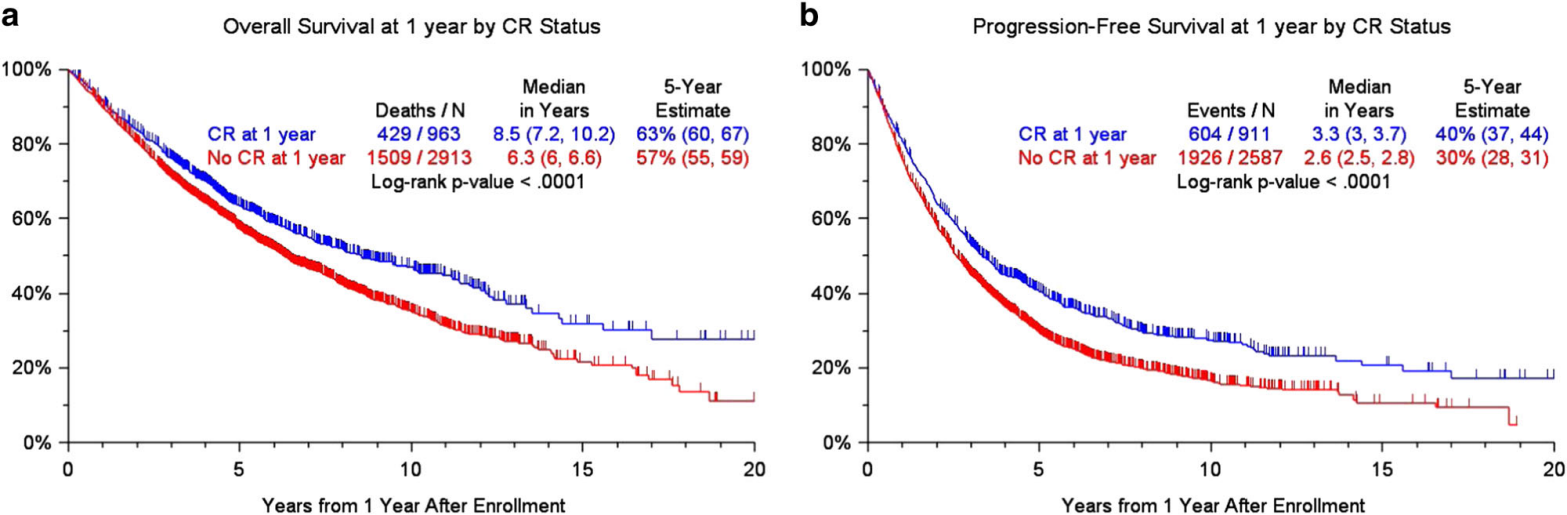
Overall survival and progression-free survival at 1 year by CR status

The OS, PFS, and CR duration separated by each country is presented in the supplemental files (Supplemental Figure 1a-d). For the combined dataset analyses, the OS and PFS were compared between patients who achieved CR landmarked at 1 year after diagnosis (Fig. 1) to examine the effects of depth of response on outcome. Patients who achieved CR had a superior PFS (median PFS 3.3 years vs. 2.6 years, *p* < 0.0001); this effect was most pronounced for the patients who received thalidomide (the only novel agent used in dataset) with higher CR rates observed and median PFS of 6 years for those who achieved CR (Supplemental Figure 1e/f). The benefit was also seen in terms of OS for the whole group (median OS 8.5 years vs. 6.3 years, *p* < 0.0001). Patients who had albumin < 3.5 g/dL (*p* = 0.002) or an IgG isotype (*p* < 0.001) were less likely to achieve a CR at the 1-year landmark (Table 1). Age over 65 years was not associated with inability to achieve CR in this dataset. We further examined clinical variables associated with sustained CR for 3 years and 5 years in subset of patients where these data were available (Supplemental Tables 6, 7) using logistic regression. These analyses were not very informative, as only IgA isotype, light chain only disease and BMPC > 30% featured with statistical significance in multivariate analyses for both time points.

**Table 1:** Patient characteristics by CR Indicator at 1 year

Factor	All patients	Less than CR within first year	CR within first year	*P*-value	Percent of patients w/ data
Age at registration> = 65 yr	258/3867 (7%)	194/2906 (7%)	64/961 (7%)	0.986	258/3867 (7%)
IgA	735/3086 (24%)	499/2326 (21%)	236/760 (31%)	< 0.001	735/3086 (24%)
IgG	1868/3086 (61%)	1549/2326 (67%)	319/760 (42%)	< 0.001	1868/3086 (61%)
Female	1380/3286 (42%)	1018/2442 (42%)	362/844 (43%)	0.542	1380/3286 (42%)
Albumin < 3.5 g/dL	1041/3443 (30%)	819/2590 (32%)	222/853 (26%)	0.002	1041/3443 (30%)
B2M > = 3.5 mg/L	1517/3681 (41%)	1126/2753 (41%)	391/928 (42%)	0.510	1517/3681 (41%)
B2M > 5.5 mg/L	708/3681 (19%)	517/2753 (19%)	191/928 (21%)	0.231	708/3681 (19%)
Creatinine > = 2 mg/dL	397/3802 (10%)	282/2852 (10%)	115/950 (12%)	0.056	397/3802 (10%)
HGB < 10 g/dL	1280/3785 (34%)	973/2842 (34%)	307/943 (33%)	0.343	1280/3785 (34%)
LDH > Upper Limit Normal	403/2244 (18%)	288/1740 (17%)	115/504 (23%)	0.002	403/2244 (18%)
Platelet Count < 150 × 10^9/L	635/3758 (17%)	454/2838 (16%)	181/920 (20%)	0.011	635/3758 (17%)
Any clonal abnormality by cytogenetics	322/1256 (26%)	209/822 (25%)	113/434 (26%)	0.814	322/1256 (26%)
BMPC > = 30%	1986/3077 (65%)	1472/2260 (65%)	514/817 (63%)	0.257	1986/3077 (65%)
ISS Stage 1	1860/2645 (70%)	1351/1949 (69%)	509/696 (73%)	0.057	1860/2645 (70%)
ISS Stage 2	1505/3302 (46%)	1121/2475 (45%)	384/827 (46%)	0.569	1505/3302 (46%)
ISS Stage 3	1242/3388 (37%)	954/2542 (38%)	288/846 (34%)	0.067	1242/3388 (37%)

*n*/*N* (%): *n*—Number with factor, *N*—Number with valid data for factor

*ND* No valid observations for factor

*P-value from Fisher’s exact test, otherwise chi-squared test. *P*-values represent a comparison between groups, not against the overall population

We also examined the clinical variables associated with 10-year survival compared with 2-year death (Table 2). In a univariate model, age ≤ 65 years (OR 2.24, *p* < 0.001), normal albumin (OR 2.01, *p* < 0.001), low beta 2 microglobulin ≤ 3.5 mg/dl (OR 3.51, *p* < 0.001), serum creatinine levels ≤ 2 mg/dL (OR 3.73, *p* < 0.001), hemoglobin levels ≥ 10 g/dL (OR 2.98, *p* < 0.001), platelet count ≥ 150k/μL (OR 3.94, *p* < 0.001), normal serum LDH levels (OR 2.78, *p* < 0.001), no cytogenetic abnormality (OR 3.21, *p* < 0.001), BM plasmacytosis < 30% (OR 2.31, *p* < 0.001), and ISS stage I or II (OR 3.59, *p* < 0.001) appeared to be positively associated with 10-year survival. In multivariate analysis, age ≤ 65 years (OR 1.87, *p* = 0.002), non-IgA Isotype (OR 1.53, *p* = 0.004), normal albumin < 3.5 g/dL (OR = 1.36, *p* = 0.023), low beta 2 microglobulin ≤ 3.5 mg/dl (OR 1.86, *p* < 0.001), serum creatinine levels < 2 mg/dL (OR 1.77, *p* = 0.005), hemoglobin levels ≥ 10 g/dL (OR 1.55, *p* = 0.003), and platelet count ≥ 150k/μL (OR 2.26, *p* < 0.001) appeared to positively associated with 10-year survival. Interestingly, cytogenetic abnormalities did not feature in multivariate analysis.

**Table 2:** Logistic regression, 10-year survival vs. 2-year death

	Factors differentiating 10-year survival vs. 2-year death				
Variable	*N*	Survival less than 2 years	Survival more than 10 years	OR (95% CI)	*P*-value
Univariate					
Age at registration > = 65 yr	2374	163/214 (76%)	1271/2160 (59%)	2.24 (1.61, 3.10)	< 0.001
IgA	1878	306/463 (66%)	785/1415 (55%)	1.56 (1.26, 1.95)	< 0.001
IgG	1878	580/1080 (54%)	511/798 (64%)	0.65 (0.54, 0.79)	< 0.001
Female	1918	493/827 (60%)	685/1091 (63%)	0.87 (0.73, 1.05)	0.157
Albumin < 3.5 g/dL	2206	559/808 (69%)	737/1398 (53%)	2.01 (1.68, 2.42)	< 0.001
B2M > = 3.5 mg/L	2208	833/1123 (74%)	488/1085 (45%)	3.51 (2.94, 4.20)	< 0.001
B2M > 5.5 mg/L	2208	501/630 (80%)	820/1578 (52%)	3.59 (2.89, 4.46)	< 0.001
Creatinine > = 2 mg/dL	2320	353/430 (82%)	1042/1890 (55%)	3.73 (2.87, 4.85)	< 0.001
HGB < 10 g/dL	2313	678/908 (75%)	699/1405 (50%)	2.98 (2.48, 3.57)	< 0.001
LDH > Upper Limit Normal	935	196/260 (75%)	354/675 (52%)	2.78 (2.02, 3.82)	< 0.001
Platelet Count < 150 × 10^9/L	2241	364/443 (82%)	969/1798 (54%)	3.94 (3.04, 5.11)	< 0.001
Any clonal abnormality by cytogenetics	507	92/145 (63%)	127/362 (35%)	3.21 (2.15, 4.80)	< 0.001
BMPC > = 30%	1933	800/1226 (65%)	317/707 (45%)	2.31 (1.91, 2.79)	< 0.001
ISS Stage 1	2070	296/761 (39%)	910/1309 (70%)	0.28 (0.23, 0.34)	< 0.001
ISS Stage 2	2105	477/756 (63%)	759/1349 (56%)	1.33 (1.11, 1.60)	0.002
ISS Stage 3	2208	501/630 (80%)	820/1578 (52%)	3.59 (2.89, 4.46)	< 0.001
Multivariate (stratified by country)					
Age at registration > = 65 yr	1230	98/144 (68%)	525/1086 (48%)	1.87 (1.26, 2.79)	0.002
IgA	1230	180/298 (60%)	443/932 (48%)	1.53 (1.15, 2.04)	0.004
Albumin < 3.5 g/dL	1230	294/474 (62%)	329/756 (44%)	1.36 (1.04, 1.78)	0.023
B2M > = 3.5 mg/L	1230	376/569 (66%)	247/661 (37%)	1.86 (1.41, 2.45)	< 0.001
Creatinine > = 2 mg/dL	1230	130/174 (75%)	493/1056 (47%)	1.77 (1.18, 2.65)	0.005
HGB < 10 g/dL	1230	288/429 (67%)	335/801 (42%)	1.55 (1.16, 2.06)	0.003
Platelet Count < 150 × 10^9/L	1230	163/218 (75%)	460/1012 (45%)	2.26 (1.59, 3.22)	< 0.001

*P*-value from Wald chi-square test in logistic regression. NS2–Multivariate results not statistically significant at 0.05 level. Univariate *p*-values reported regardless of significance. Multivariate model uses stepwise selection with entry level 0.1 and variable remains if meets the 0.05 level. A multivariate *p*-value greater than 0.05 indicates variable forced into model with significant variables chosen using stepwise selection

*OR* odds ratio, *95% CI* 95% confidence interval

**Fig. 2:**
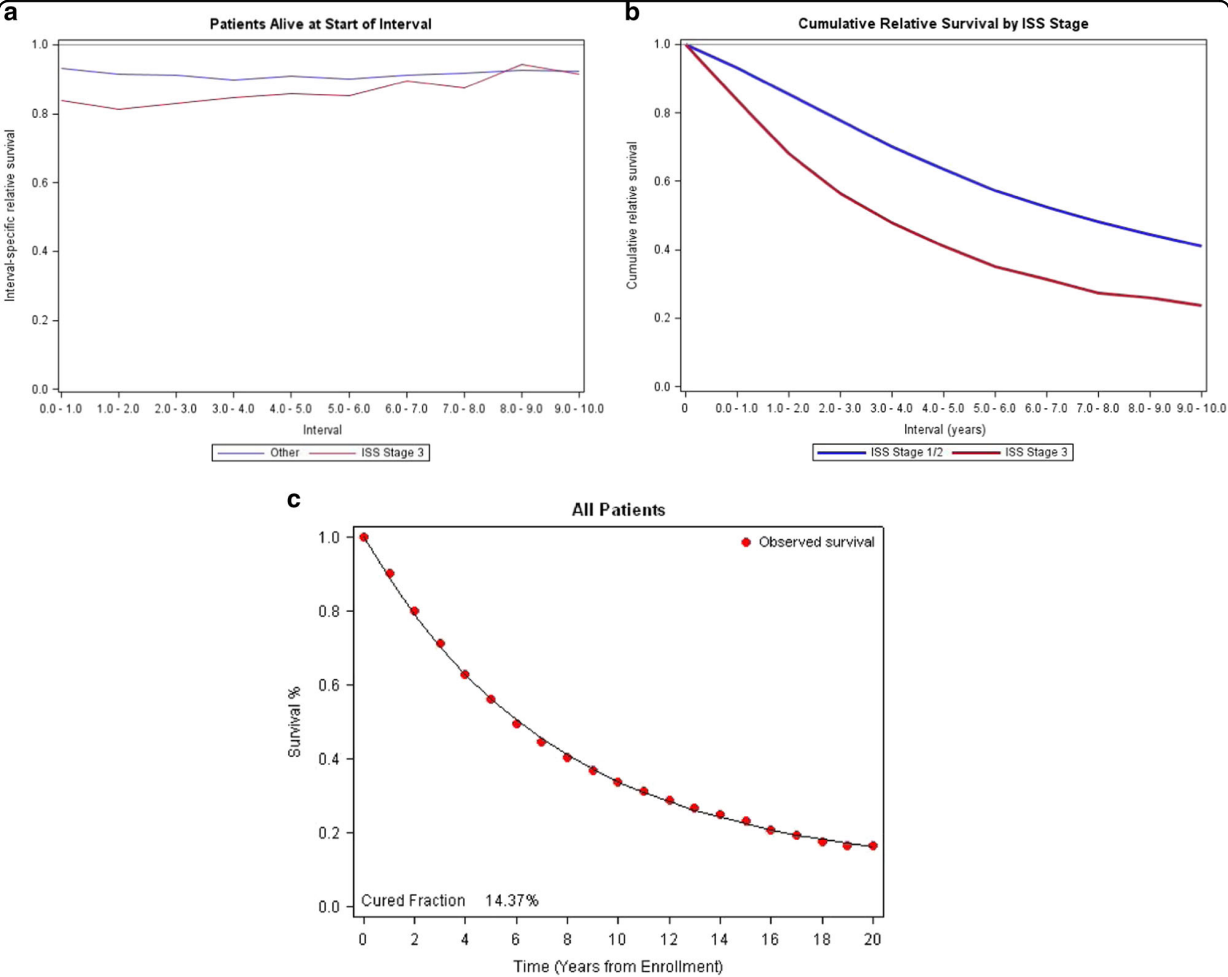
Relative Survival Analyses (A) Relative survival, ISS Stage III vs. ISS Stage I/II, (B) Cumulative relative survival, ISS Stage III vs. ISS Stage I/II, (C) Cure fraction for the study population

We then examined the RS of the whole patient cohort to matched general population. We note that RS was between 0.8 and 0.95 for all countries during the first several years of therapy, with RS for all countries combined close to 0.9, the ISS III patients had a lower RS compared to ISS I/II patients (Fig. 2a). Cumulative RS plots by ISS staging also shows a decline over time in the ISS III patients’ probability of achieving RS similar to matched population (Fig. 2b). The RS curves were also examined by country (Supplemental Figures 3a-h) and showed similar trends to the overall dataset. The statistical cure fraction for the whole group appears to be 14.3% (Fig. 2c), which signifies the overall proportion of MM patients in this cohort who were able to achieve or exceed expected survival compared to matched general population.

## Discussion

There have been several large population based studies that have highlighted the trends of improvements in MM survival and outcomes^11–14^. Whereas we can attribute this success to the therapeutic success of new drug classes, it also clear that not every MM patient achieves the same depth of response. We also appreciate that despite achieving a good depth of by current IMWG criteria, a proportion of MM patients remains at a high risk of relapse within the first 2 years of diagnosis^15^. It has also been observed that, despite not achieving CR, patients with documented antecedent smoldering course and those presenting with a MGUS-like gene expression profiling (GEP) signature can have OS of over 10 years (75% patients) after HDM-ASCT^16^. This perhaps explains the subgroup of PR patients in the Spanish Myeloma group experience that remain progression free at 12-years^7^.

Several next generation sequencing studies in MM^17,18^ have elegantly demonstrated the heterogeneous nature of MM, even within the same patient during the course of disease, when treated sequentially with novel therapies. These data highlight the genetic chaos that is present from the very onset of disease and further evolves rapidly with each relapse so that eventually total resistance to salvage interventions ensues. We also have to appreciate that there are perhaps more effective drugs and drug classes in the clinician’s armamentarium than was available for MM patients being treated in the 1990s or even early 2000s. This may mean that the depth of response after induction therapy may continue to improve over time, potentially further improving the PFS/OS of biologic subset who previously achieved PR yet had good long-term survival.

Cure modeling has been utilized for survival analysis for over three decades. In general, survival analyses include censored observations for subjects who are lost to follow-up or have not experienced and event at the time the analysis is conducted. Most cure modeling has been done in pediatric cancer population, where the other causes of death can be ignored on account of rarity. In case of adult cancer, there may be subjects who never have disease recurrence and perhaps have been cured of the disease. Cure modeling is a special statistical technique which attempts to capture this subset of patients and several different techniques such as cure fractions and RS rate have been described in literature^19^. To calculate cure fraction, one has to assume that the disease is cured and that it is difficult to make this technique applicable to the whole MM population due to competing causes of death—but perhaps the younger, HDM-ASCT eligible MM patients may benefit from such an analysis^20^. In the present analysis, we see a cure fraction of 14.37% for the study population which is impressive; yet we must bear in mind that this is a select, HDM-ASCT eligible MM patient population on clinical trials. It is important to emphasize that, in order to have a mature long-term follow-up, most patients included in the present study were treated with conventional agents before the era of novel drugs.

To adjust for the lack of evidence of cure for individual patients, a better approach may be looking at “statistical” or “functional” cure via RS modeling. This latter technique compares the expected survival of a given subjects with similar characteristics and co-morbidities to the actual survival as a result of cancer. The expected survival is readily available through life tables available through national mortality statistics. For most cancers, it takes several years before the RS curve reach a plateau. We observe a similar trend for HDM-ASCT eligible MM patients in the current analysis, where there is steady rise in the RS beyond 5 years of diagnosis regardless of ISS staging.

The present study is the largest cohort of HDM-ASCT eligible patients that has been collected to interrogate the clinical predictors of long-term survivors. An important caveat in the current study is that the dataset includes HDM-ASCT eligible MM patients enrolled on clinical trials. Choosing this cohort afforded us completeness of baseline clinical variables, availability of long-term follow-up and survival data. We identified achieving CR in the first year of diagnosis an important landmark as it was found to be associated with superior PFS and OS. This is an important finding and underscores the importance of depth of response as we explore novel regimens for newly diagnosed MM along with MRD endpoints. Next, the study identified older age at diagnosis and higher burden of disease (by BM plasmacytosis, level of anemia and serum creatinine levels, etc.) adversely affected probability of long-term survival ( > 10 years) compared with early death ( < 2 years). Although high risk features such as elevated serum LDH levels and having any cytogenetics abnormalities were adverse variables in univariate analysis, they did not feature in multivariate analysis. This perhaps is a function of having inadequate cytogenetics information on all the patients in the dataset and reliance on conventional band karyotyping.

## Electronic supplementary material

Supplementary Figures and Tables
