# Supplementary material for: Clinical predictors of long-term survival in newly diagnosed transplant eligible multiple myeloma — an IMWG Research Project
Source: Blood Cancer J. 2018 Nov 23;8(12):123. doi: 10.1038/s41408-018-0155-7 (PMC6251924; doi:10.1038/s41408-018-0155-7)
Supplement: Supplementary file 1 — Supplementary Figures and Tables [file 41408_2018_155_MOESM1_ESM.docx]

**SUPPLEMENTAL TABLES/FIGURES**

**Figure 1a: OS/PFS/CRD, all patients**

**Figure 1b: OS/PFS separated by country**

|  | OS | PFS |
| --- | --- | --- |
| Czech Republic |  |  |
| France |  |  |
| Germany |  |  |
| Italy |  |  |
| Korea |  |  |
| Spain |  |  |
| Nordic |  |  |
| USA (MI) |  |  |
| USA (SWOG) |  |  |

**Figure 1c: CRD by country**

|  | CRD |
| --- | --- |
| Czech Republic |  |
| France |  |
| Germany |  |
| Italy |  |
| Korea |  |
| USA (MI) |  |
| USA (SWOG) |  |

**Supplemental Figure 1d: OS, PFS by presence/absence of novel agents**

**Supplemental Figure 1e: PFS by CR at 1 year, patients receiving novel agent(s)**

**Supplemental Figure 1f: PFS by CR at 1 year, patients receiving no novel agents**

**Supplemental Figure 2a: Relative survival, Czech Republic, all patients with relative survival data**


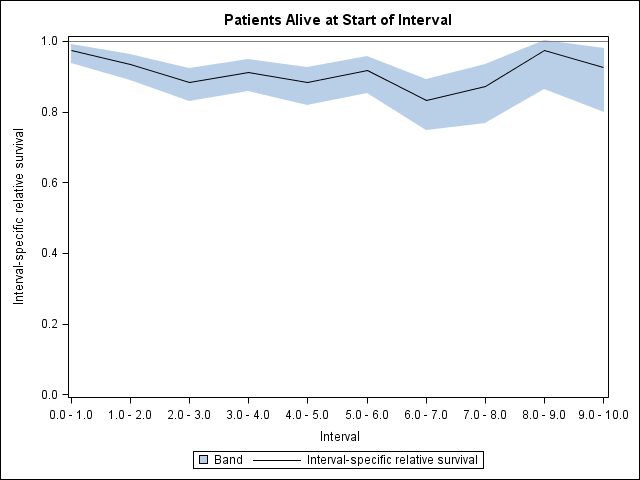


**Supplemental Figure 2b: Relative survival, France, all patients with relative survival data**


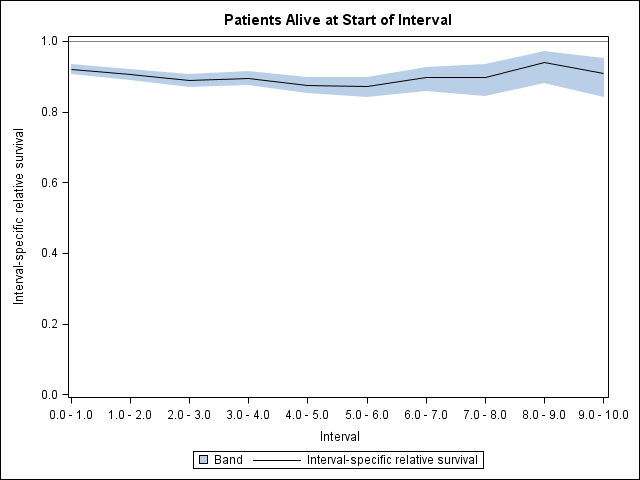


**Supplemental Figure 2c: Relative survival, Germany, all patients with relative survival data**


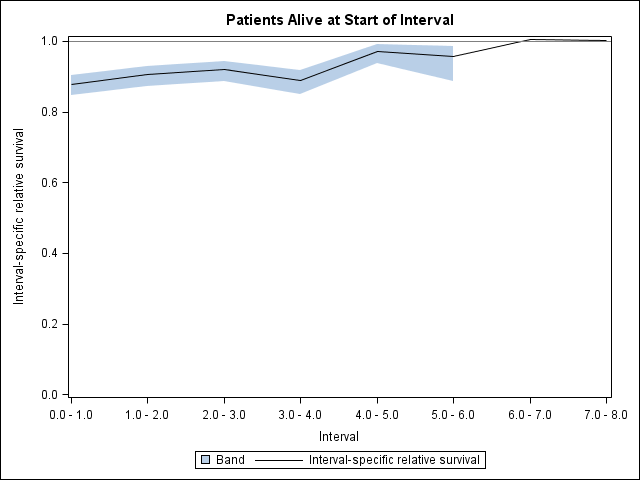


**Supplemental Figure 2d: Relative survival, Italy, all patients with relative survival data**

**
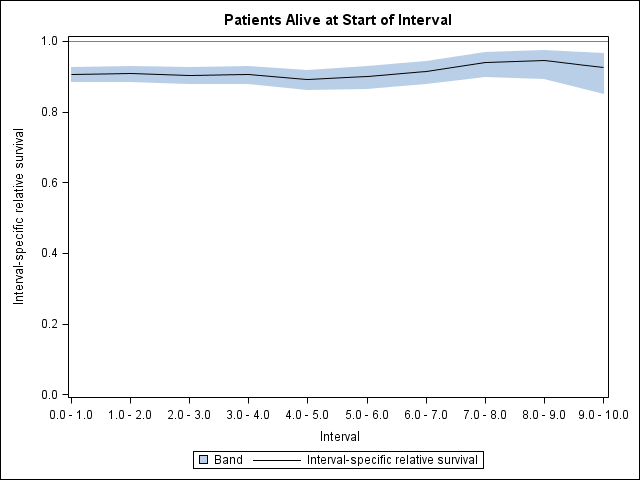
**

**Supplemental Figure 2e: Relative survival, Korea, all patients with relative survival data**


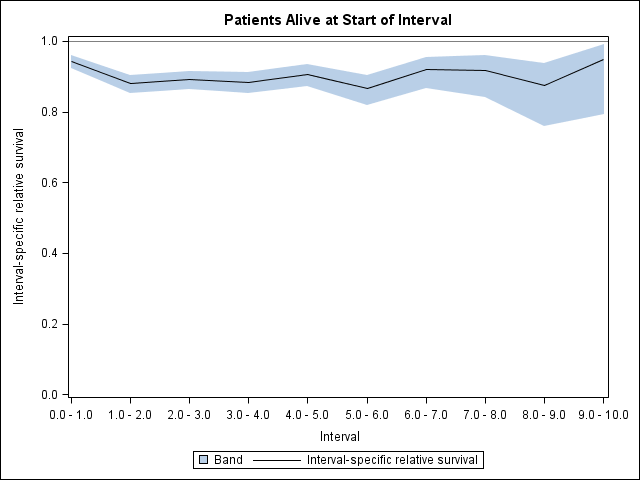


**Supplemental Figure 2f: Relative survival, Spain, all patients with relative survival data**

**
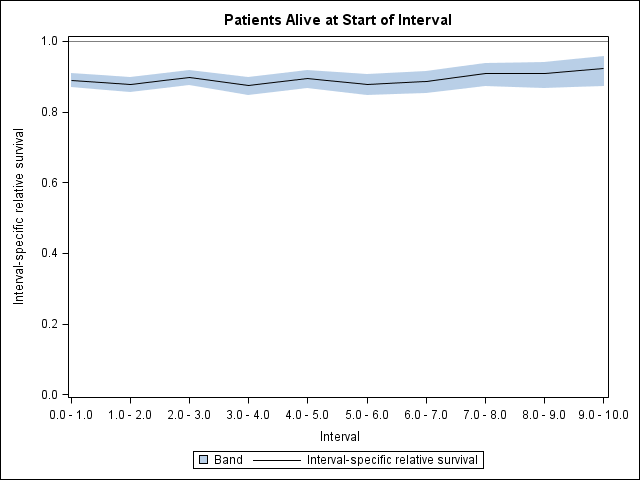
**

**Supplemental Figure 2g: Relative survival, USA (MI), all patients with relative survival data**


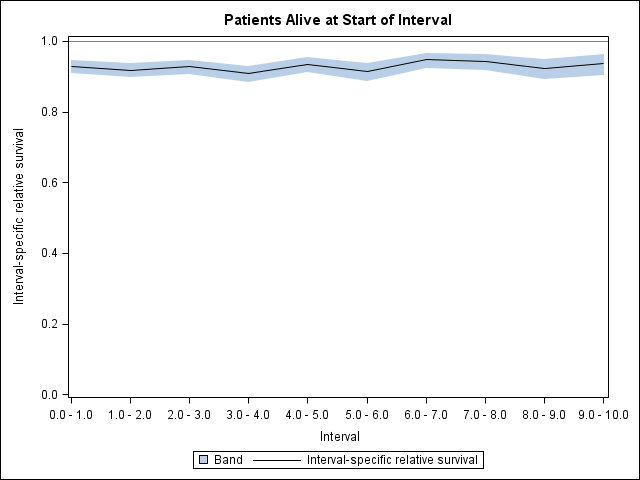


**Supplemental Figure 2h: Relative survival, USA (SWOG), all patients with relative survival data**


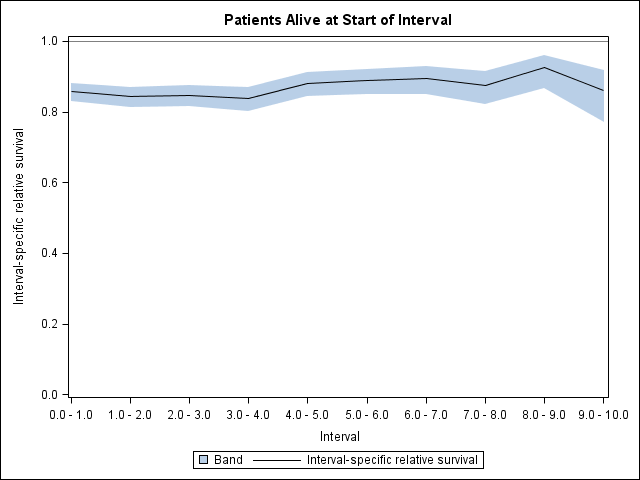


**Supplemental Table 1: Table of long-term follow-up by country**

|  | Median  follow-  up (years) | 5-year follow-up | 6-year follow-up | 7-year follow-up | 8-year follow-up | 9-year follow-up | 10-year follow-up | 12-year follow-up | 15-year follow-up |
| --- | --- | --- | --- | --- | --- | --- | --- | --- | --- |
| Czech | 11.1  (8.0, 12.2) | 137 (61.4%) | 123  (55.2%) | 81  (36.3%) | 52  (23.3%) | 48  (21.5%) | 41  (18.4%) | 28  (12.6%) | 7  (3.1%) |
| France | 6.4  (6.3, 6.6) | 662 (44.3%) | 428  (28.7%) | 242  (16.2%) | 133  (8.9%) | 122  (8.2%) | 109  (7.3%) | 58  (3.9%) | 10  (0.7%) |
| Germany | 4.9  (4.7, 5.0) | 149 (27.9%) | 38  (7.1%) | 1  (0.2%) | 0  (0.0%) | 0  (0.0%) | 0  (0.0%) | 0  (0.0%) | 0  (0.0%) |
| Italy | 8.2  (7.8, 8.5) | 392 (54.0%) | 327  (45.0%) | 243  (33.5%) | 171  (23.6%) | 111  (15.3%) | 77  (10.6%) | 42  (5.8) | 2  (0.3%) |
| Korea | 6.1  (5.8, 6.4) | 302 (39.8%) | 197  (26.0%) | 124  (16.3%) | 70  (9.2%) | 44  (5.8%) | 23  (3.0%) | 8  (1.6%) | 2  (0.3%) |
| Spain | 9.7  (9.4, 9.9) | 514 (47.5%) | 434  (40.1%) | 362  (33.4%) | 298  (27.5%) | 212  (19.6%) | 139  (12.8%) | 16  (1.5%) | 0  (0.0%) |
| Nordic (Sweden, Denmark, Norway) | 16.6  (16.3, 17.1) | 410 (54.4%) | 351 (46.6%) | 304 (40.3%) | 268 (35.5%) | 241 (32.0%) | 201 (26.7%) | 152 (20.2%) | 112 (14.9%) |
| USA (MI) | 11.5  (11.2, 12.0) | 575 (64.0%) | 519  (57.7%) | 484  (53.8%) | 443  (49.3%) | 379  (42.2%) | 286  (31.8%) | 149  (16.6%) | 54  (6.01%) |
| USA (SWOG) | 9.1  (8.7, 9.5) | 344  (42.2%) | 285  (34.9%) | 227  (27.8%) | 162  (19.9%) | 111  (13.6%) | 65  (8.0%) | 19  (2.3%) | 4  (0.5%) |

**Supplemental Table 2: Table of early deaths by country**

|  | 1-year early death | 2-year early death |
| --- | --- | --- |
| Czech | 8 (3.6%) | 24 (10.8%) |
| France | 129 (8.7%) | 263 (17.6%) |
| Germany | 68 (12.7%) | 114 (21.3%) |
| Italy | 71 (9.8%) | 134 (18.5%) |
| Korea | 45 (5.9%) | 131 (17.3%) |
| Spain | 126 (11.6%) | 247 (22.8%) |
| Nordic (Sweden, Denmark, Norway) | 74 (9.8%) | 140 (18.6%) |
| USA (MI) | 71 (7.9%) | 146 (16.2%) |
| USA (SWOG) | 122  (15.0%) | 235  (28.0%) |

**Supplemental Table 3: Summary of major missing data by country (patients with survival data)**

| **Factor** | **All Patients** | **Czech Republic** | **France** | **Germany** | **Italy** | **Korea** | **Spain** | **Nordic (Sweden, Denmark, Norway)** | **USA (MI)** | **USA (SWOG)** |
| --- | --- | --- | --- | --- | --- | --- | --- | --- | --- | --- |
| Age | 10/7161 (0%) | 0/222 (0%) | 10/1408 (1%) | 0/532 (0%) | 0/723 (0%) | 0/750 (0%) | 0/1076 (0%) | 0/738 (0%) | 0/896 (0%) | 0/816 (0%) |
| Sex | 1623/7161 (23%) | 0/222 (0%) | 885/1408 (63%) | 0/532 (0%) | 0/723 (0%) | 0/750 (0%) | 0/1076 (0%) | 738/738 (100%) | 0/896 (0%) | 0/816 (0%) |
| Heavy chain | 1780/7161 (25%) | 0/222 (0%) | 1408/1408 (100%) | 13/532 (2%) | 1/723 (0%) | 181/750 (24%) | 2/1076 (0%) | 141/738 (19%) | 0/896 (0%) | 34/816 (4%) |
| Albumin | 676/7161 (9%) | 8/222 (4%) | 110/1408 (8%) | 61/532 (11%) | 369/723 (51%) | 15/750 (2%) | 48/1076 (4%) | 45/738 (6%) | 4/896 (0%) | 16/816 (2%) |
| B2M | 488/7161 (7%) | 12/222 (5%) | 30/1408 (2%) | 107/532 (20%) | 49/723 (7%) | 35/750 (5%) | 69/1076 (6%) | 138/738 (19%) | 2/896 (0%) | 46/816 (6%) |
| Creatinine | 179/7161 (2%) | 13/222 (6%) | 100/1408 (7%) | 10/532 (2%) | 0/723 (0%) | 14/750 (2%) | 14/1076 (1%) | 4/738 (1%) | 14/896 (2%) | 10/816 (1%) |
| HGB | 189/7161 (3%) | 0/222 (0%) | 68/1408 (5%) | 90/532 (17%) | 0/723 (0%) | 12/750 (2%) | 10/1076 (1%) | 1/738 (0%) | 1/896 (0%) | 7/816 (1%) |
| Platelets | 337/7161 (5%) | 0/222 (0%) | 229/1408 (16%) | 5/532 (1%) | 1/723 (0%) | 71/750 (9%) | 19/1076 (2%) | 3/738 (0%) | 1/896 (0%) | 8/816 (1%) |
| LDH, ULN but No Value | 770/7161 (11%) | 4/222 (2%) | 0/1408 (0%) | 3/532 (1%) | 19/723 (3%) | 0/750 (0%) | 0/1076 (0%) | 738/738 (100%) | 3/896 (0%) | 3/816 (0%) |
| LDH, Value but No UNL | 1858/7161 (26%) | 50/222 (23%) | 1141/1408 (81%) | 7/532 (1%) | 0/723 (0%) | 655/750 (87%) | 0/1076 (0%) | 0/738 (0%) | 0/896 (0%) | 5/816 (1%) |
| LDH, No Value nor ULN | 1858/7161 (26%) | 5/222 (2%) | 267/1408 (19%) | 12/532 (2%) | 369/723 (51%) | 95/750 (13%) | 1076/1076 (100%) | 0/738 (0%) | 0/896 (0%) | 34/816 (4%) |
| BMPC% | 1227/7161 (17%) | 0/222 (0%) | 132/1408 (9%) | 45/532 (8%) | 9/723 (1%) | 66/750 (9%) | 16/1076 (1%) | 19/738 (3%) | 124/896 (14%) | 816/816 (100%) |
| Any clonal abnormality by cytogenetics | 5739/7161 (80%) | 147/222 (66%) | 1408/1408 (100%) | 532/532 (100%) | 723/723 (100%) | 270/750 (36%) | 1076/1076 (100%) | 738/738 (100%) | 29/896 (3%) | 816/816 (100%) |
| Bone lesions | 2149/7161 (30%) | 2/222 (1%) | 1408/1408 (100%) | 400/532 (75%) | 0/723 (0%) | 46/750 (6%) | 19/1076 (2%) | 4/738 (1%) | 259/896 (29%) | 11/816 (1%) |
| n/N (%):  n- Number with factor, N- Number with valid data for factor ND: No valid observations for factor | | | | | | | | | | |

**Supplemental Table 4: Annotation of all therapies by country**

| **Country** | **Institution** | **Therapy** | **Novel agents?** |
| --- | --- | --- | --- |
| Czech  Republic | Czech Myeloma Group (CMG) | 4W – VAD+TX+UL Interferon  4W – VAD+TX+UL Interferon+Dex  CMG2002 – VAD+TX+UL Interferon  CMG2002 – Other | N  N  N  N |
| France | IFM | IFM90: BMT arm  IFM90: Chemo arm  IFM94  IFM9902  IFM9904 | N  N  N  Unknown  Unknown |
| Germany | University of Heidelberg | GMMG-HD3, Reference arm  GMMG-HD3, Thalidomide arm | N  Y |
| Italy | University of Bologna | BO1996 – Single ASCT  BO1996 – Double ASCT  BO2002 – TD | N  N  Y |
| Korea | KMMWP | VAD  M+Pd | N  N |
| Spain | University of Salamanca | GEM2000 | N |
| Nordic (Sweden, Denmark, Norway) | NMSG | 5/94, 7/98 Trials | N |
| USA (MIRT) | Myeloma Institute for Research and Therapy | TT1  TT2: Non-thalidomide arm  TT2: Thalidomide arm | N  N  Y |
| USA (SWOG) | SWOG | S9321 | N |

**Supplemental Table 5: Patient Characteristics by Presence/Absence of Novel Agents**

| **Factor** | **All Patients** | **No novel agents** | **Novel agent(s)** | **P-value** |
| --- | --- | --- | --- | --- |
| Age at registration >= 65 yr | 591/6276 (9%) | 499/5330 (9%) | 92/946 (10%) | 0.726 |
| IgA | 1333/5381 (25%) | 1101/4441 (25%) | 232/940 (25%) | 0.943 |
| IgG | 3190/5381 (59%) | 2648/4441 (60%) | 542/940 (58%) | 0.266 |
| Female | 2347/5538 (42%) | 1939/4592 (42%) | 408/946 (43%) | 0.609 |
| **Albumin < 3.5 g/dL** | **2019/5689 (35%)** | **1887/5129 (37%)** | **132/560 (24%)** | **<.001** |
| **B2M >= 3.5 mg/L** | **2646/5797 (46%)** | **2308/4941 (47%)** | **338/856 (39%)** | **<.001** |
| **B2M > 5.5 mg/L** | **1317/5797 (23%)** | **1164/4941 (24%)** | **153/856 (18%)** | **<.001** |
| **Creatinine >= 2 mg/dL** | **834/6188 (13%)** | **740/5254 (14%)** | **94/934 (10%)** | **<.001** |
| **HGB < 10 g/dL** | **2312/6150 (38%)** | **2024/5249 (39%)** | **288/901 (32%)** | **<.001** |
| LDH > Upper Limit Normal | 555/2675 (21%) | 363/1765 (21%) | 192/910 (21%) | 0.748 |
| **Platelet Count < 150 x 10^9/L** | **1110/6006 (18%)** | **974/5064 (19%)** | **136/942 (14%)** | **<.001** |
| Any clonal abnormality by cytogenetics | 372/1422 (26%) | 279/1100 (25%) | 93/322 (29%) | 0.210 |
| **BMPC >= 30%** | **3330/5148 (65%)** | **2667/4305 (62%)** | **663/843 (79%)** | **<.001** |
| **At least 1 bone lesion** | **4101/5012 (82%)** | **3632/4277 (85%)** | **469/735 (64%)** | **<.001** |
| **ISS Stage 1** | **2110/5311 (40%)** | **1841/4801 (38%)** | **269/510 (53%)** | **<.001** |
| ISS Stage 2 | 2097/5426 (39%) | 1862/4836 (39%) | 235/590 (40%) | 0.532 |
| **ISS Stage 3** | **1317/5797 (23%)** | **1164/4941 (24%)** | **153/856 (18%)** | **<.001** |
| n/N (%):  n- Number with factor, N- Number with valid data for factor ND: No valid observations for factor * P-value from Fisher's exact test, otherwise chi-squared test. P-values represent a comparison between groups, not against the overall population. | | | | |

**Supplemental Table 6: Logistic regression for sustained CR vs. no sustained CR, patients with 3-year survival and available data for sustained CR**

|  | | **Factors differentiating 3-yr survival with sustained CR vs. without sustained CR** | | | | |
| --- | --- | --- | --- | --- | --- | --- |
|  | **Variable** | **N** | **With Factor** | **Without Factor** | **OR (95% CI)** | **P - value** |
| Univariate | Age at registration >= 65 yr | 3064 | 68/197 (35%) | 840/2867 (29%) | 0.95 (0.69, 1.32) | 0.765 |
|  | **IgA** | **2419** | **180/541 (33%)** | **533/1878 (28%)** | **1.27 (1.03, 1.58)** | **0.025** |
|  | **IgG** | **2419** | **369/1502 (25%)** | **344/917 (38%)** | **0.55 (0.46, 0.66)** | **<0.001** |
|  | **Light Chain Only** | **2419** | **124/289 (43%)** | **589/2130 (28%)** | **2.19 (1.68, 2.86)** | **<0.001** |
|  | **Other (heavy chain)** | **2419** | **164/376 (44%)** | **549/2043 (27%)** | **2.04 (1.61, 2.59)** | **<0.001** |
|  | Female | 2573 | 341/1088 (31%) | 439/1485 (30%) | 1.09 (0.92, 1.30) | 0.318 |
|  | **Albumin < 3.5 g/dL** | **2727** | **182/765 (24%)** | **613/1962 (31%)** | **0.72 (0.59, 0.88)** | **0.002** |
|  | **B2M >= 3.5 mg/L** | **2927** | **297/1091 (27%)** | **586/1836 (32%)** | **0.79 (0.67, 0.94)** | **0.008** |
|  | B2M > 5.5 mg/L | 2927 | 136/464 (29%) | 747/2463 (30%) | 0.94 (0.75, 1.18) | 0.577 |
|  | Creatinine >= 2 mg/dL | 3012 | 86/250 (34%) | 809/2762 (29%) | 1.25 (0.94, 1.66) | 0.123 |
|  | HGB < 10 g/dL | 3004 | 264/940 (28%) | 621/2064 (30%) | 0.91 (0.76, 1.08) | 0.281 |
|  | LDH > Upper Limit Normal | 1782 | 112/291 (38%) | 435/1491 (29%) | 1.25 (0.95, 1.66) | 0.109 |
|  | Platelet Count < 150 x 10^9/L | 2988 | 128/433 (30%) | 758/2555 (30%) | 0.84 (0.66, 1.06) | 0.135 |
|  | Any clonal abnormality by cytogenetics | 1017 | 88/223 (39%) | 326/794 (41%) | 0.85 (0.62, 1.15) | 0.284 |
|  | **BMPC >= 30%** | **2480** | **464/1557 (30%)** | **319/923 (35%)** | **0.77 (0.64, 0.93)** | **0.006** |
|  | **ISS Stage 1** | **2623** | **422/1299 (32%)** | **356/1324 (27%)** | **1.27 (1.06, 1.52)** | **0.008** |
|  | **ISS Stage 2** | **2688** | **250/971 (26%)** | **543/1717 (32%)** | **0.82 (0.68, 0.98)** | **0.030** |
|  | ISS Stage 3 | 2927 | 136/464 (29%) | 747/2463 (30%) | 0.94 (0.75, 1.18) | 0.577 |
| Multivariate (stratified by country) | **IgA (vs. IgG)** | **1877** | **153/424 (36%)** | **442/1453 (30%)** | **1.55 (1.21, 1.97)** | **<.001** |
|  | **Light Chain Only (vs. IgG)** | **1877** | **95/215 (44%)** | **500/1662 (30%)** | **2.29 (1.68, 3.12)** | **<.001** |
|  | Other (vs. IgG) | 1877 | 127/287 (44%) | 468/1590 (29%) | 1.61 (0.98, 2.64) | 0.060 |
|  | **BMPC >= 30%** | **1877** | **387/1278 (30%)** | **208/599 (35%)** | **0.76 (0.61, 0.95)** | **0.017** |
| **OR - Odds Ratio, 95% CI - 95% Confidence Interval, P - value from Wald Chi - Square Test in Logistic Regression NS2 - Multivariate results not statistically significant at 0.05 level. Univariate p - values reported regardless of significance. Multivariate model uses stepwise selection with entry level 0.1 and variable remains if meets the 0.05 level. A multivariate p - value greater than 0.05 indicates variable forced into model with significant variables chosen using stepwise selection.** | | | | | | |

**Supplemental Table 7: Logistic regression for sustained CR vs. no sustained CR, patients with 5-year survival and available data for sustained CR**

|  | | **Factors differentiating 5-yr survival with sustained CR vs. without sustained CR** | | | | |
| --- | --- | --- | --- | --- | --- | --- |
|  | **Variable** | **N** | **With Factor** | **Without Factor** | **OR (95% CI)** | **P - value** |
| Univariate | Age at registration >= 65 yr | 2116 | 49/137 (36%) | 539/1979 (27%) | 0.99 (0.67, 1.46) | 0.971 |
|  | **IgA** | 1702 | 113/364 (31%) | 385/1338 (29%) | 1.16 (0.89, 1.50) | 0.276 |
|  | **IgG** | **1702** | **272/1071 (25%)** | **226/631 (36%)** | **0.63 (0.50, 0.78)** | **<0.001** |
|  | **Light Chain Only** | **1702** | **84/202 (42%)** | **414/1500 (28%)** | **2.03 (1.47, 2.80)** | **<0.001** |
|  | **Other (heavy chain)** | **1702** | **113/267 (42%)** | **385/1435 (27%)** | **1.85 (1.39, 2.46)** | **<0.001** |
|  | Female | 1795 | 236/784 (30%) | 293/1011 (29%) | 1.08 (0.87, 1.33) | 0.479 |
|  | **Albumin < 3.5 g/dL** | **1871** | **102/484 (21%)** | **413/1387 (30%)** | **0.70 (0.54, 0.91)** | **0.007** |
|  | B2M > 3.5 mg/L | 2035 | 180/703 (26%) | 398/1332 (30%) | 0.83 (0.67, 1.03) | 0.083 |
|  | B2M > 5.5 mg/L | 2035 | 78/299 (26%) | 500/1736 (29%) | 0.90 (0.67, 1.20) | 0.473 |
|  | Creatinine >= 2 mg/dL | 2082 | 51/159 (32%) | 526/1923 (27%) | 1.37 (0.95, 1.98) | 0.089 |
|  | HGB < 10 g/dL | 2099 | 157/606 (26%) | 422/1493 (28%) | 0.93 (0.74, 1.16) | 0.523 |
|  | LDH > Upper Limit Normal | 1281 | 79/201 (39%) | 321/1080 (30%) | 1.24 (0.89, 1.72) | 0.208 |
|  | Platelet Count < 150 x 10^9/L | 2071 | 75/260 (29%) | 502/1811 (28%) | 0.91 (0.67, 1.24) | 0.559 |
|  | **Any clonal abnormality by cytogenetics** | **755** | **47/150 (31%)** | **255/605 (42%)** | **0.56 (0.38, 0.83)** | **0.003** |
|  | **BMPC >= 30%** | **1691** | **286/1041 (27%)** | **212/650 (33%)** | **0.70 (0.55, 0.88)** | **0.002** |
|  | **ISS Stage 1** | **1812** | **299/954 (31%)** | **212/858 (25%)** | **1.28 (1.03, 1.60)** | **0.025** |
|  | **ISS Stage 2** | **1853** | **150/633 (24%)** | **371/1220 (30%)** | **0.79 (0.62, 0.99)** | **0.040** |
|  | ISS Stage 3 | 2035 | 78/299 (26%) | 500/1736 (29%) | 0.90 (0.67, 1.20) | 0.473 |
| Multivariate (stratified by country) | **IgA (vs. IgG)** | **1303** | **94/278 (34%)** | **317/1025 (31%)** | **1.37 (1.01, 1.84)** | **0.040** |
|  | **Light Chain Only (vs. IgG)** | **1303** | **65/146 (45%)** | **346/1157 (30%)** | **2.09 (1.44, 3.02)** | **<0.001** |
|  | Other (vs. IgG) | 1303 | 88/200 (44%) | 323/1103 (29%) | 1.47 (0.82, 2.61) | 0.193 |
|  | **BMPC >= 30%** | **1303** | **251/860 (29%)** | **160/443 (36%)** | **0.67 (0.52, 0.88)** | **0.003** |
| **OR - Odds Ratio, 95% CI - 95% Confidence Interval, P - value from Wald Chi - Square Test in Logistic Regression NS2 - Multivariate results not statistically significant at 0.05 level. Univariate p - values reported regardless of significance. Multivariate model uses stepwise selection with entry level 0.1 and variable remains if meets the 0.05 level. A multivariate p - value greater than 0.05 indicates variable forced into model with significant variables chosen using stepwise selection.** | | | | | | |
